# A Hospital of All the Virtues

**Published:** 1889-12-28

**Authors:** 


### Fallow Crops.

### A HOSPITAL OF ALL THE VIRTUES.

Among our acquaintance we all reckon certain people for whom nothing seems quite good enough. Perhaps they are young and ardent, or it may be they are old and peevish. But though they be unlike in all else, in this they agree—they are dissatisfied with what is, and, entirely dissenting from Pope's optimistic philosophy, they would fain amend his conclusions, and say, "whatever is, is wrong." Moreover, they are firmly convinced that everything will remain in that unhappy condition until such time as they have been allowed to put it right, and thus they may be likened to living and unappreciated prototypes of those pills and plaisters of universal application, which, if only mankind at large could be got to take freely to them, would eradicate all the ills flesh is heir to, and pain and disease would cease to be anything but memories.

A section of these well-meaning, but slightly egotistical people, is now attempting to organise its forces for a descent upon our system of medical education and relief, and the world is promised a hospital "conducted upon anti-vivisectional principles," to which those who wish to do good may subscribe without fear "that they may be in reality encouraging evil."

It is one of the curiosities of our boasted liberty that a hospital may be started by anybody, whether it is wanted or not, and may be conducted upon any principles whatever, or, for that matter, upon no principles at all. It is to the latter condition we fear the proposed institution must come. For if principle be a sacred fundamental truth, it is obvious there can be no principle when that we seek to display as truth rests upon and is inseparable from what we stigmatise as error. When the promoters of the proposed hospital announce that "the treatment employed will be in accordance with the highest medical and surgical knowledge of the time" they must be perfectly well aware that this promise can only be honestly performed by abandoning the principle they ostentatiously avow, and which supplies the sole *raison d'être* of the proposed establishment.

If "thoroughly qualified physicians and surgeons will be appointed," these gentlemen must have acquired their qualifications in the very schools denounced by the authors of the prospectus we are considering. It is not necessary that they should have actually performed vivisection. On the contrary, it is a part of the oft reiterated answer to the charges somewhat unscrupulously repeated that there is no such general practice of vivisection as is implied by the sweeping assertions retailed by its adversaries. None the less "the highest medical and surgical skill of the time" is unattainable except through sources which, we are assured, are enriched by its aid. This may or may not be satisfactory, but it is an insoluble fact, and one of which medical education cannot be purged so long as the profession, as represented by its leaders and instructors, is practically unanimous.

It is to these considerations that we must look for an explanation of the poverty of the professional following this embryo hospital boasts. The "clique of physiologists" of which the prospectus makes pathetic mention is evidently potent enough to exact obedience or sagacious enough to induce conviction. The faculty has made up its mind, and the opposition of a few professional dissenters is no more to the purpose than the somewhat arbitrary dictum of a famous philanthropist which surmounts the prospectus by way of motto. Lord Shaftesbury's horror of vivisection was born of a noble tenderness of heart, but the assertion he is said to have made that "vivisection is an abominable thing, and hateful in the sight of God," lends no strength to the efforts for the establishment of the hospital it is proposed to call by his name. The probability is that whatever the strength of the late lord's hatred of the practice of vivisection, one so determinedly opposed to shams would have refused his countenance to a project set forth in terms alike remarkable for the boldness of the premises and the weakness of the conclusions.

It would appear but a feeble ambition which seeks "to provide an institution to which the generous may subscribe without fear of doing harm"—we should have thought these words applicable to many institutions already existing—but when the aspiration is translated into a hope to strike a blow "at cruel and unscientific methods"—an anticipation that the proposed hospital "will cure a larger proportion of patients in a shorter time than usual," and a confident prediction that "the heart of the nation" will not withhold its sympathy, it is disappointing to find that the utmost result looked for is "to make a start in a private house with a few beds." Surely those who wish to do good without fear "that they may be in reality encouraging evil" should be capable of something more than this! If we accept implicitly the statements in this prospectus, we must believe that they are a numerous class restrained from exercising their philanthropic instincts by the enormities of our existing hospitals.

We take leave, however, to doubt the actuality of this multitude of non-contents, and to protest against a wholesale calumny of our noblest institutions. As to the question of vivisection pure and simple, this is scarcely the place to discuss it. But far be it from us, who have recently shown ourselves fully alive to the importance of maintaining unsullied the benevolent and religious character of our hospitals, to limit our humanity to mankind. We will agree that vivisection, if it be not capable of great good, is not justifiable, and that in any case the enactments controlling its practice must be strictly upheld and possibly rendered more stringent. But a great deal more than the question of vivisection would be raised by a serious effort to establish a hospital upon the principles enunciated by the promoters of the scheme under notice. Agricola.

